# Prevalence, predictors and severity of xerostomia in adult patients in general dental care

**DOI:** 10.3389/froh.2026.1795172

**Published:** 2026-06-01

**Authors:** Anna Adolfsson, Frida Lenér, Annica Almståhl, Ulrica Almhöjd, Bertil Marklund, Hülya Çevik-Aras

**Affiliations:** 1 Department of Oral Medicine and Pathology, Institute of Odontology, University of Gothenburg, Gothenburg, Sweden; 2 Specialist Clinic of Orofacial Medicine Uddevalla/Trollhättan, Trollhättan, Sweden; 3 Region Västra Götaland, Research, Education, Development & Innovation (REDI), Primary Health Care, Gothenburg, Sweden; 4 Department of Psychiatry and Neurochemistry, Institute of Neuroscience and Physiology, Sahlgrenska Academy, University of Gothenburg, Gothenburg, Sweden; 5 Department of Microbiology and Immunology, Institute of Odontology, University of Gothenburg, Gothenburg, Sweden; 6 Department of Oral Health, Faculty of Odontology, Malmö University, Malmö, Sweden; 7 Chalmers University of Technology, Gothenburg, Sweden; 8 Section of Oral Biology and Immunopathology/Oral Medicine, Department of Odontology, Faculty of Health and Medical Sciences, University of Copenhagen, Copenhagen, Denmark

**Keywords:** adult population, dry mouth, medication, oral health, polypharmacy, salivary hypofunction, xerostomia

## Abstract

**Objectives:**

While xerostomia (subjective dry mouth) is frequently studied in elderly populations, this study focuses on adults aged 18–82 years to explore prevalence across a broader age spectrum, including younger adults who may experience xerostomia due to medications or systemic conditions. The objective of this study was to determine the prevalence and severity of xerostomia in patients visiting general dental care, and to assess whether age, sex, comorbidities, and medications are associated with the condition.

**Methods:**

This cross-sectional study included adult patients (aged ≥ 18 years) visiting two general dental clinics in Region Västra Götaland in Sweden. Patients agreeing to participate were asked, “Have you experienced dry mouth in the last six months?”. Participants replying “Yes” filled in the 11-item questionnaire Xerostomia Inventory to determine variability and severity of symptoms. Participants reporting no experience of dry mouth the last six months were included in the non-xerostomia group.

**Results:**

Out of 344 participants, the prevalence of xerostomia was 34.6% (*n* = 119). Self-reported use of ≥1 medication was the strongest predictor (OR: 2.50, 95% CI 1.42–4.29, *p* = 0.001) of xerostomia. Xerostomia was reported by 45.6% of participants with at least one disease compared with 31.2% of those without reported disease. Age, and biological sex, were not significant predictors.

**Conclusion and clinical relevance:**

The prevalence of xerostomia was high among patients seeking general dental care. Since early diagnosis and preventive dental care are crucial for managing the condition, awareness of the association between medications and xerostomia could benefit dental and health care professionals, ultimately improving patient outcomes.

## 1 Introduction

Saliva plays crucial roles in various physiological processes including lubrication, facilitating taste perception, aiding in the chewing and swallowing of food, and speech. Whole saliva is mainly composed of water (∼99.5%), along with proteins, minerals, microorganisms, and cells (1). It also contains buffer systems that stabilises pH in the oral cavity, and antimicrobial proteins that contribute to the innate immune defence against pathogens (1–4).

The subjective variant of dry mouth, xerostomia, is the condition defined as the individual's perceived symptom of having a dry mouth. It may be linked to changes in salivary flow, however these changes are not always associated with a reduction in volume (5). Common symptoms of xerostomia include issues with speech, chewing, and swallowing. People suffering from xerostomia may also suffer from bad breath, a burning sensation in the mouth, an increase of dental caries, oral mucosal changes, and greater susceptibility to oral fungal infections. In addition to physiological issues, xerostomia is associated with a decrease in overall well-being and reduced oral-health-related quality of life (6–10).

Xerostomia can occur as a side effect of medication use, where the risk increases with number of prescribed medications (11–14). Dry mouth is closely related to various systemic and autoimmune diseases, including diabetes, viral infections, thyroid disease and sarcoidosis. Neurodegenerative diseases such as Parkinson's and Alzheimer's have also been associated with xerostomia with or without reduced saliva production (15, 16). Additionally, patients with Sjögren's disease and those who have undergone radiation therapy to the head and neck frequently report dry mouth (15, 17, 18). Other causes of dry mouth include dehydration, nutrient deficiencies, fibromyalgia, febrile conditions, depression, anxiety, stress, and smoking (16, 19, 20). It is also frequently associated with burning mouth syndrome (21).

The reported prevalence of xerostomia varies widely, from 0.9% to 64.8% in studies that include both subjective (xerostomia) and objective (hyposalivation) measures of oral dryness. Previous studies have reported higher prevalence in women at 10%–33%, compared to men at 10%–26% (22, 23). These substantial variations are likely due to differences in age of populations studied which mainly included participants from 50 years of age to the elderly, and the differing methods used to assess oral dryness (22–24). A majority of previous studies have focused on investigating the prevalence of xerostomia among older adults, possibly due to its association with deteriorating oral health-related quality of life in the aging population (25). However, the prevalence of xerostomia in adults across the whole age range from 18 years has not previously been studied, except in our previous study conducted in primary health care (26). In the aforementioned study we have obtained a high xerostomia prevalence of 43.6%. Additionally, use of medication was a significant predictor of xerostomia, regardless of age and biological sex. Notably, 71.2% of patients with polypharmacy (≥5 medications) presented the highest prevalence of xerostomia. The results obtained in the primary care setting represents a study population with high medication use and a high burden of systemic diseases. However, the general dental care setting in Sweden more closely represents a normal adult population, as traditionally a large part of the adult population visits general dental care for annual or biannual check-ups, while primary care visits are scheduled when individuals present a medical need. Hence our hypothesis is that the prevalence of xerostomia among patients in general dental care will be lower than in primary care, reflecting differences in medication use and comorbidities.

The current study focused on adults aged ≥18 years attending general dental care providers in the Västra Götaland Region (VGR), Sweden, with the aim of determining the prevalence and severity of xerostomia, and examining its association with age ranges, sex, comorbidities, and medication use.

## 2 Materials and methods

The Swedish Ethical Review Board approved the study (reg. no. 2021-04213). All participants signed an informed consent form prior to inclusion in the study.

### 2.1 Study population

This cross-sectional study was carried out among patients aged ≥18–82 years, visiting two general dental care clinics in Region Västra Götaland for any medical reason during January to July 2021. Patients were asked to participate by an entrance host and receptionist. Participants lacking capacity to consent, e.g., not understanding the language or exhibiting cognitive impairment, were excluded. Out of the 432 patients invited to participate, 45 declined and 42 were excluded during data analysis due to missing information in the surveys, for example due to missing to sign the informed consent or not answering all the 11 statements in the Xerostomia inventory (XI) questionnaire.

Patients agreeing to participate completed a printed questionnaire in which they were asked to self-report demographic and health-related information, including age, biological sex, diseases, and daily medication use. Disease and medication burden were each assessed using two sequential questions: participants first indicated yes or no to “Do you have any diseases?” and “Do you take any medication on a daily basis?”, and if affirmative, provided a numerical estimate.

The participants were thereafter asked to answer the initial screening question; “Have you experienced dry mouth in the last six months?” A total of 119 participants replied “Yes” and proceeded to fill in the Swedish translation of the 11-item Xerostomia Inventory (XI) questionnaire to determine the variability and severity of symptoms. Each item has 5 answering alternatives: “Never”, “Rarely”, “Sometimes”, “Often”, and “Always”. The total Likert scale score was calculated from the participants' responses, ranging between 11 and 55. A higher score indicates multiple and/or more severe symptoms. Participants who had not experienced symptoms of dry mouth in the last six months (*n* = 225) were included in the non-xerostomia group. One participant who reported “other” as gender, have been excluded from the regression analysis.

### 2.2 Data analysis

Continuous data were presented as mean ± standard deviation (SD) and nominal or categorical data as absolute and relative frequencies. All statistical analyses were performed using IBM SPSS Statistics for Windows, version 29 (IBM Corp., Armonk, N.Y., USA). Participants with xerostomia were divided into three groups according to the total score from the XI questionnaire: Mild xerostomia (12–22 points), moderate xerostomia (23–33 points), and severe xerostomia (34–49 points) (26).

A logistic regression model with forced entry method was conducted with xerostomia as a dependent variable and age, sex, reported use of ≥1 medicine and reported ≥1 disease as predictor variables. Age was set as a continuous variable, while sex, medication use, and disease status were coded as categorical variables. Simple logistic regression was performed prior to the model, with each predictor described independently. Spearman's correlation was performed among all predictor variables to assess multicollinearity prior to entry into the model. Due to the cross-sectional design of the study, the results were presented as Odds Ratio (OR) with 95% confidence interval (CI). *P*-values < 0.05 were considered statistically significant.

According to our sample size calculation with the estimated prevalence of xerostomia of 20% and a 95% confidence interval (CI) with a 5% margin of error, *n* = 264 patients needed to be included (assuming Clopper-Pearson for conservative reasons). The proportions test was performed with One-sample binomial test in IBM SPSS. Single power value was set to 0.95, null value to 0.5, and population proportion to 0.2.

## 3 Results

### 3.1 Sample description

Table 1 presents the demographic data of all participants (*n* = 344) stratified by xerostomia (*n* = 119) and non-xerostomia (*n* = 225). Age (continuous variable) ranged from 18 to 82 years with a mean of 45.5 ± 19.3 years. The mean age among female participants was 44.7 years, and the mean age among male participants was 46.5 years (data not shown). Smoking was reported by 20 participants (5.8%), out of which 7 had xerostomia and 13 did not.

**Table 1:** Demographic characteristics of participants (*n* = 344) stratified by self-reported xerostomia status. Absolute (ƒ) and relative frequencies of total (%) of xerostomia is distinguished across four age groups. Total *n* may vary across variables due to missing responses in individual questionnaire sections.

	All patients	Xerostomia	Non-xerostomia
*n* (%)	344	119 (34.6%)	225 (65.4%)
Sex			
Females; *n* (%)	196 (56.9%)	74 (62.2%)	122 (53.9%)
Males; *n* (%)	148 (43.1%)	45 (37.8%)	103 (45.6%)
Age, years: mean ± SD	45.5 ± 19.3	46.7 ± 19.6	44.9 ± 19.1
Age 18–35 years: ƒ (%)	141 (40.9%)	51 (14.8%)	90 (26.2%)
Age 36–55 years: ƒ (%)	79 (22.9%)	22 (6.4%)	57 (16.6%)
Age 56–70 years: ƒ (%)	86 (25%)	30 (8.7%)	56 (16.3%)
Age 71–82 years: ƒ (%)	38 (11.1%)	16 (4.7%)	22 (6.5%)
Any disease			
Yes; *n* (%)	79 (22.9%)	36 (30.3%)	43 (19%)
No; *n* (%)	266 (77.1%)	83 (69.7%)	183 (81%)
≥1 medicine			
Yes; *n* (%)	154 (44.6%)	70 (58.8%)	84 (37.2%)
No; *n* (%)	191 (55.4%)	49 (41.2%)	142 (62.8%)
Smoking; *n* (%)	20 (5.8%)	7 (5.9%)	13 (5.8%)

The overall prevalence of xerostomia among the 344 general dental care patients was 34.6%. The mean age was similar between the xerostomia group (46.7 ± 19.6 years) and the non-xerostomia group (44.9 ± 19.1 years). The prevalence of xerostomia tended to be higher in females (37.8%) compared to males (30.4%), though this difference was not statistically significant. Xerostomia was reported by 45.5% (*n* = 70/154) of participants using ≥1 medicine, compared to 25.8% (*n* = 49/190) of those reporting no medication use. Among the 79 participants reporting at least one disease, 45.6% (*n* = 36/79) reported xerostomia, compared to 31.2% (*n* = 83/266) of those without any reported disease.

### 3.2 Predictors of xerostomia

As shown in Table 2, participants with self-reported use of ≥1 medication had 2.50 times higher odds of xerostomia compared to those without medication use (OR: 2.50, 95% CI: 1.42–4.29, *p* = 0.001) after controlling for age, sex and presence of ≥1 self-reported disease. Although self-reported presence of ≥1 disease was significantly associated with xerostomia in simple logistic regression (OR: 1.89, 95% CI: 1.13–3.16, *p* = 0.015), this association did not remain significant in the multivariable model (OR: 1.14, 95% CI: 0.62–2.08, *p* = 0.673). Age and sex were not statistically significant predictors in either model.

**Table 2:** Simple and multivariable logistic regression analyses of predictors of xerostomia. Total *n* may vary across variables due to missing responses in individual questionnaire sections. CI, confidence interval.

	Univariate logistic regression per independent variables			Forced entry logistic regression model of selected variables. *n* = 343	
Independent variables	*n*	*p*-value	Odds Ratio (95% CI)	*p*-value	Odds Ratio (95% CI)
Age	344	0.399	1.01 (0.99–1.02)	0.597	1.00 (0.98–1.01)
Sex	344	0.157	1.39 (0.88–2.19)	0.136	1.43 (0.89–2.30)
≥1 medicine	343	<0.001	2.42 (1.54–3.82)	0.001	2.50 (1.42–4.29)
Number of medicines	335	0.007	1.17 (1.04–1.30)	–	–
≥1 disease	344	0.015	1.89 (1.13–3.16)	0.673	1.14 (0.62–2.08)
Number of diseases	338	0.001	1.59 (1.20–2.10)	–	–

### 3.3 Severity and symptoms

Of the 119 participants reporting xerostomia, the majority (48.7%, *n* = 58) had an XI-score of 23–33 points, placing them in the group “moderate xerostomia”, whereas 36.1% (*n* = 43) experienced mild symptoms and 15.1% (*n* = 18) severe symptoms.

There was a skew towards a higher XI-score among females compared to males.

Table 3 portrays the severity of specified symptoms for participants reporting dry mouth. Out of the symptoms the participants ranked “Rarely” or more frequent in the XI questionnaire, the most reported one was having dry lips (96.6%) followed by dry skin (92.4%), and dry eyes (83.2%). The most common intraoral symptom was “I wake at night to drink water” (79.8%). Moreover, 50.4% of the participants with xerostomia felt intraoral dryness while eating, and 56.3% needed to drink to enable swallowing. 41.2% reported that they struggled with swallowing specific kinds of foods, and especially dry food items (52.9%). To relieve symptoms of xerostomia 47.1% used/needed to use lozenges or chewing gum. Among participants with xerostomia, 75.6% also reported dryness of the nasal mucosa.

**Table 3:** The severity of xerostomia symptoms according to stated assumptions in the questionnaire. Graded on a likert scale.

Assumptions	Never	Rarely	Sometimes	Often	Always	%
My mouth feels dry	0.8	19.3	57.1	16.0	6.7	
I get up at night to drink water	19.3	29.4	29.4	16.8	4.2	
My mouth is dry when I eat	49.6	32.8	11.8	4.2	1.7	
I need to drink to be able to swallow food	43.7	31.1	17.6	5.9	1.7	
It is difficult to swallow certain foods	58.8	26.1	14.3		0.8	
It is difficult to eat dry food	42.9	28.6	17.6	5.9	0.8	
I use/need to use lozenges or gum to alleviate dry mouth	52.1	13.4	21.0	8.4	4.2	
My skin feels dry	7.6	13.4	42.0	25.2	11.8	
My eyes feel dry	16.8	20.2	41.2	16.0	5.9	
My lips feel dry	3.4	13.4	45.4	30.3	7.6	
The inside of my nose feels dry	24.4	29.4	28.6	14.3	3.4	

## 4 Discussion

This study found a xerostomia prevalence of 34.6% among adults aged ≥18 years seeking general dental care. The mean age in the xerostomia group was similar (46.7 years) to that in the non-xerostomia group (44.9 years), and age was not a statistically significant predictor for xerostomia. Females presented a higher prevalence of xerostomia at 37.8%, compared to males at 30.4%. The prevalence of 34.6% in this study was lower compared to our previous study which found a prevalence of 43.6% among patients in primary health care (26). The current study possibly reflects a prevalence closer to the general population compared to data collected in primary care, as individuals seeking primary care are often more likely to have a multiple health conditions and/or medications than those seeking general dental care. In the current study, 44.8% of the participants reported using medication, a lower proportion compared to primary care, where nearly 70% used medication (26). Furthermore, in the region where this study was conducted, financial aid and social structures enable adults from most social and economic backgrounds to visit dental clinics regularly. Therefore, we expected the prevalence in the current study to be closer to the previously reported value in the general population, approximately 20% (27).

Previous studies highlight increasing age as a risk factor for xerostomia (22–24). Ohara et al. (28) describes how physical frailty in older adults is linked to xerostomia but not necessarily to hyposalivation. The prevalence of xerostomia in the elderly population is associated with medication use (29–34), and we emphasize the importance of distinguishing between a variable being “associated with” vs. “contributing to” xerostomia, due to the inherent difference of correlation and causation.

In terms of differences between the sexes, studies generally report that women are at a higher risk of xerostomia (22–25), which is consistent with our findings. However, sex was not a significant predictor in the present study. It is likely that females are physiologically susceptible to dry mouth conditions. For example, during menopause hormonal changes may promote changes in the salivary glands, potentially leading to xerostomia. The reasons why females prior to menopause are at higher risk of developing xerostomia are not yet known.

The strong association between medication use and xerostomia found in this study aligns with previous reports on medication-induced xerostomia (15, 16). The systematic review from World Workshop on Oral Medicine VI (22) and a recent study (35) reported that a wide range of drugs can cause xerostomia including those affecting the nervous, cardiovascular, genitourinary, musculoskeletal, respiratory, and alimentary systems. The side effects of medications on salivary glands vary, some target by sympathomimetic or anticholinergic pathways, while diuretics might impair the ion-transporting channels in acinar cells. The latter may cause xerostomia due to the dehydration effect (35). Medications can inhibit the secretory pathway of major and minor salivary glands at multiple levels (14, 29, 32).

Previous research has shown a relationship between some kinds of smoking and dry mouth conditions; however, we did not notice an effect of smoking in our study group. This may be due to the low number of smokers.

Despite the high prevalence of xerostomia and the negative consequences of a missed diagnosis, needs are often unmet (36). For the general dental practitioner, it is valuable to consider the extent of this debilitating condition. Fisic et al. (36) recently reported that the clinical awareness and management of xerostomia and its complications is not well recognized in general dental care. Their study shows that older adults were more often asked about oral dryness and its symptoms than young patients. Despite the known correlation between xerostomia and decreasing oral health, only 30.5% of dentists and 42.5% of dental hygienists recommended preventive dental care (36). These results bring prominence to our study, as expanding awareness of the condition and its management by dental and health care professionals is crucial.

Saliva is fundamental in maintaining a healthy homeostasis of the oral cavity. Individuals affected by a reduced salivary secretion rate and/or xerostomia experience a decrease in health-related quality of life (6–8). Reporting on prevalence of xerostomia found in this study can help raise awareness among dental health practitioners, emphasizing the clinical significance of recognizing the strong correlation between medication use and xerostomia.

To reduce xerostomia, multiple strategies should be considered. These include educating the patient of measurements such as maintaining optimal hydration, good oral hygiene, fluorides, and administering saliva substitutes and/or stimulants. Regular dental check-ups to monitor and manage complications of xerostomia should be advised. The possibility to discuss substitution of drugs with xerostomia as side-effect with the patients’ physician could be considered. Despite current knowledge of xerostomia, dental care providers may overlook the diagnosis (22), which leads to insufficient dental care. Early detection and alleviation of symptoms may have significant impacts on quality of life and overall physical health. Considering this, a well-established collaboration between primary healthcare providers and dental care professionals would greatly benefit individuals with xerostomia.

A limitation of the study is that the specific types of diseases and medications used by participants were not registered due to logistical and ethical limitations. This information was furthermore assessed through simple self-report, and the possibility that participants may have missed to fill in their correct information should be considered.

Analyzing medication categories would require a substantially larger study population to achieve adequate statistical power, which was not feasible within the present study design. For these reasons, medication categories were not included. However, our previous register-based study showed that medications used for the treatment of metabolic and alimentary tract diseases (16.07%), nervous system disorders (16.04%), and cardiovascular diseases (12.63%) were associated with the highest percentage of xerostomia diagnosis (35).

The study population consisted of adults aged 18 years and older visiting general dental care in Sweden, which reflects heterogeneity in terms of age, health status, and medication use. However, all data was collected through self-reported questionnaires, which may increase the risk for under- or over-reporting of symptoms and conditions.

Another limitation of the study worth mentioning is that the cost of dental care in Sweden is usually not subsidized for adults after age of 23 in VGR, which might have limited the number of participants with low socioeconomic status.

The assessment methods mostly used in the general dental clinic are single- or multi-item questionnaires, such as the Xerostomia Inventory with 11 questions measuring variability and severity of subjective dry mouth (23). In the current study a non-validated but previously used Swedish translation of the validated English version of Xerostomia Inventory (XI) was used as the assessment method for xerostomia (26, 37). By using such an instrument, one loses some degree of subjectivity since the questionnaire has no open-ended questions. For example, a participant having severe and debilitating xerostomia symptoms while eating and swallowing, or a participant with constantly disturbed sleep due to lack of unstimulated whole saliva during the night, may render a low XI-score, if the individual experiences no other symptoms.

## 5 Conclusions

The prevalence of xerostomia was 34.6% among patients seeking general dental care. Of the variables included in the multivariable model (age, sex, medication use, and presence of disease), only medication use remained significantly associated with xerostomia. Increased awareness of the side effects of medications and their association with xerostomia would benefit dental and health care professionals, ultimately improving patient outcomes. Early diagnosis and preventive dental care are crucial clinical implications for managing the condition.

## Data Availability

The data that support the findings of this study are available on request from the corresponding author. The data are not publicly available due to ethical restrictions.
