# Correction: Prevalence, predictors and severity of xerostomia in adult patients in general dental care

**DOI:** 10.3389/froh.2026.1917692

**Published:** 2026-07-07

**Authors:** Anna Adolfsson, Frida Lenér, Annica Almståhl, Ulrica Almhöjd, Bertil Marklund, Hülya Çevik-Aras

**Affiliations:** 1 Department of Oral Medicine and Pathology, Institute of Odontology, University of Gothenburg, Gothenburg, Sweden; 2 Specialist Clinic of Orofacial Medicine Uddevalla/Trollhättan, Trollhättan, Sweden; 3 Region Västra Götaland, Research, Education, Development & Innovation (REDI), Primary Health Care, Gothenburg, Sweden; 4 Department of Psychiatry and Neurochemistry, Institute of Neuroscience and Physiology, Sahlgrenska Academy, University of Gothenburg, Gothenburg, Sweden; 5 Department of Microbiology and Immunology, Institute of Odontology, University of Gothenburg, Gothenburg, Sweden; 6 Department of Oral Health, Faculty of Odontology, Malmö University, Malmö, Sweden; 7 Chalmers University of Technology, Gothenburg, Sweden; 8 Section of Oral Biology and Immunopathology/Oral Medicine, Department of Odontology, Faculty of Health and Medical Sciences, University of Copenhagen, Copenhagen, Denmark

**Keywords:** adult population, dry mouth, medication, oral health, polypharmacy, salivary hypofunction, xerostomia

The Data Availability statement was erroneously given as ‘The raw data supporting the conclusions of this article will be made available by the authors, without undue reservation.’

The correct Data Availability statement is ‘The data that support the findings of this study are available on request from the corresponding author. The data are not publicly available due to ethical restrictions.’

The ethics statement was erroneously given as ‘All members are part of the Swedish Ethical Review Authority-committee Magnus ForsbergÅsa HannaRonnie LundströmPeter AppelrosHugo LövheimJenny PerssonUlrika Pettersson KymmerSven Arne SilfverdalThomas BrännströmMikael Sandlund Per-Erik Lundmark Christina Snell Lumio Lars-Erik Olofsson Mats DahlströmAnn-Britt GrünewaldInger Trodell DahlAgne FuringstenKristina ArnrupLars-Gunnar GunnarssonYvonne Freund-LeviMartin HöglundMargareta MöllerOlof ErikssonArja Harila-SaariMårten FryknäsJaneth Leksell. The studies were conducted in accordance with the local legislation and institutional requirements. The participants provided their written informed consent to participate in this study.’

The correct ethics statement is ‘The studies were approved by the Swedish Ethical Review Authority (Dnr 2020-03094 and Dnr 2021-04213) and were henceforth conducted in accordance with stated requirements. Participants provided written informed consent prior to participation.’

The Generative AI statement: States that we have used generative AI in the creation of this manuscript. We have not used any generative AI.

A correction has been made to the section Generative AI statement:

“The author(s) declared that no generative AI was used in the creation of this manuscript”

The original version of this article has been updated.

